# Perception, Reaction, and Future Development of the Influence of COVID-19 on the Hospitality and Tourism Industry in China

**DOI:** 10.3390/ijerph19020991

**Published:** 2022-01-16

**Authors:** Lina Zhong, Sunny Sun, Rob Law, Xiaonan Li, Liyu Yang

**Affiliations:** 1 Institute for Big Data Research in Tourism, School of Tourism Sciences, Beijing International Studies University, Beijing 100020, China; zhonglina@bisu.edu.cn (L.Z.); 2019110221@stu.bisu.edu.cn (X.L.); yang.liyu@outlook.com (L.Y.); 2 College of Asia Pacific Studies, Ritsumeikan Asia Pacific University, Beppu, Oita 874–8577, Japan; 3 Asia-Pacific Academy of Economics and Management, Department of Integrated Resort and Tourism Management, Faculty of Business Administration, University of Macau, Taipa, Macau 999078, China; roblaw@um.edu.mo

**Keywords:** perception, reaction, COVID-19, tourism, PRP crisis model, public health

## Abstract

The present study examined the perception, reaction (i.e., possible measures), and future development from the perspectives of hotel and tourism practitioners and experts to investigate the influence of coronavirus disease 2019 (i.e., COVID-19) on the hospitality and tourism industry in China. After conducting 58 in-depth interviews among hotel and tourism practitioners and experts, feasible and practical measures were proposed to reduce such influence and predict the future development of China’s hospitality and tourism industry. Findings indicate that the influence of COVID-19 on the industry is perceived mainly through the pandemic’s economic and social effects. Possible measures that can be adopted for the recovery of China’s hospitality and tourism industry include the following aspects: government financial support, employee relationship management and electronic (e)-training, business marketing management, and industry co-operation network. A Perception-Reaction-Predication (PRP) crisis model is also proposed.

## 1. Introduction

The current outbreak of the novel coronavirus (COVID-19) has had a tremendous effect on the hospitality and tourism industry worldwide [1]. According to Pforr [2], the tourism industry, and hotels in particular, is vulnerable to various crises and/or disasters. Examples of crises and/or disasters include economic crises, terrorism and political turmoil, disease outbreaks, and natural disasters, such as floods, hurricanes, and earthquakes [3,4]. Potential tourists may cancel their trips to a specific destination that has been hit by a crisis/disaster, which will in turn negatively affect the destination economy [5,6]. Breitsohl and Garrod [7] have reported that crises and disasters consistently have always had negative effects on the hospitality and tourism industry. For example, the severe acute respiratory syndrome (SARS) outbreak in 2003 led to a 9% loss of tourism in Asia, whereas the global tourism industry suffered losses of up to USD 16.8 billion [8]. Specifically, in early May 2003, using Marco Polo Hotel in Hong Kong as an example, its occupancy rate dropped to 10% compared with that of 83% in the same month of 2002. Similarly, occupancy rates at the Great Eagle and Eaton Hotels in Hong Kong in April and May of 2003 dropped to approximately 7%–8% compared with an average of approximately 90% in 2002 [9]. According to Chien and Law [10], in the two months following the outbreak of SARS in March, the number of hotel guests dropped to an incomparable level. SARS also affected the hospitality industry in other Asian countries, such as South Korea, and even in developed countries, such as Canada. According to Kim et al. [11], South Korea recorded its lowest hotel occupancy rate from April to June 2003. In the second quarter of 2003, the hotel industry in Canada had a $316 million loss in hotel room revenues [12]. In addition, the cancellation and postponement of meetings resulted in huge losses for the hotel industry in Toronto [13]. The outbreak of the Ebola virus in west Africa in 2014 also severely hit the hospitality industry, given that the number of tourists dropped in the affected areas in African countries [14].

Tourism products are mostly intangible and cannot be tested before purchase [15], making it difficult for potential visitors to evaluate the quality of purchased tourism products, such as advance hotel reservations [16]. Safety is always considered the most important factor compared with other factors affecting tourists’ travel decisions [6]. Thus, if tourists consider the destination to be unsafe, they will always avoid traveling to such a destination. Cavlek [17] has indicated that crises, such as infectious diseases, not only affect the tourist flow to a specific destination but also to the neighboring areas or countries. At present, COVID-19 continues to have different degrees of effects on the hospitality and tourism industry in different countries and regions worldwide [18,19,20]. International, regional, and local travel restrictions largely affect the national economy [21]. The number of tourists in the first quarter of 2020 fell by 22% (compared to the same quarter in 2019) [22]. It is also expected that the contribution of global tourism to the GDP will drop from −2.93 to −7.82 percentage points, and employment will be reduced from −2.44 to −6.55 percentage points [23]. Even after the pandemic, it may still take two to five years for international tourism to return to the pre-COVID-19 state [24].

Xu and Grunewald [25] have reported that each stakeholder needs to fully understand the corresponding strategy and resource management methods to prepare for further crises. Safety is considered a decisive factor for tourists to visit a certain destination, and thus, tourism product providers, such as hotels, must reduce the uncertainty and risk perception for consumers [16]. Based on previous literature, advance preparation [26] and effective post-disaster reconstruction, such as in-time communication, and coordination among different stakeholders [27] are considered effective methods of recovering the tourism industry in a certain destination.

In summary, although the effects of crises, such as infectious diseases, on the hospitality and tourism industry have been examined by previous studies, feasible measures to reduce the effects of infectious diseases on the industry have received limited attention. Considering the large influence of the scale of COVID-19 on the global hospitality and tourism industry, exploring how this disease affects the industry and producing effective ways to reduce its effect are of great importance. Thus, the present study investigates the influence of COVID-19 on the industry by using China as an example to provide references for other countries and regions. This study aims to identify the influence of COVID-19 on the hospitality and tourism industry, propose practical and effective measures to reduce such influence, and predict future development of the hospitality and tourism industry affected by COVID-19 from the perspective of hotel and tourism experts and practitioners.

## 2. Literature Review

### 2.1. Effects of Infectious Diseases on the Hospitality and Tourism Industry

Based on existing literature, infectious diseases have had a considerable influence on hospitality and tourism [7]. For example, in 2003 SARS had severely hit Hong Kong’s hotel industry, and the average occupancy rate of Hong Kong Kowloon hotels dropped to approximately 10% during the Easter holidays compared to 70–80% in the previous year [9]. Chien and Law [10] studied the effect of SARS on the hotel industry of Hong Kong by identifying risks and providing suggestions for the industry in case of similar situations in the future. Henderson and Ng [28] outlined the influence of SARS on Singapore’s hotel industry, such as the drop in hotel occupancy rate, the decrease in business and leisure travelers, and the large number of unemployed people. Lee and Warner [29] believed that SARS would have a medium-term or even a long-term effect on people’s psychology. Wu, et al. [30] found that the global outbreak of influenza A (H1N1) negatively affected the hospitality industry, which was reflected in the low hotel occupancy rate caused by a drop in tourist arrivals. The recent Ebola virus in 2014 severely hit tourism in many African countries, as Baker [14] pointed out that tourists were afraid of visiting any of the African countries.

Previous studies developed different research models to investigate the effects of crises/disasters on the hospitality and tourism industry. For example, Hayes and Patton [27] developed a design methodology to implement a proactive crisis management strategy. Their strategic approach was composed of four stages, namely, strategic level, risk assessment, crisis management organization and operations, and crisis resolution and recovery. Keogh-Brown and Smith [31] designed an economic evaluation model to analyze the influence of SARS on affected economies and found that the actual influence of such an outbreak on the economy was less than that of contemporary media reports and model estimates. Hystad and Keller [32] developed a crisis management model and found that marketing strategies and communication and coordination with emergency organizations are considered important tasks for tourism organizations to consider. Hystad and Keller [32] and De Sausmarez [33] divided crisis and disaster management into three phases, namely, pre-, mid-, and post-crisis phases. Racherla and Hu [34] also strengthened the importance of a three-stage crisis management framework that includes pre-crisis, crisis, and post-crisis stages. Given the current situation of the COVID-19 pandemic worldwide, this study focused on the crisis and post-crisis stages to investigate the influence of such a pandemic on the hospitality industry.

### 2.2. Crisis Recovery Measures

#### 2.2.1. Advance Preparation

Several studies indicated the importance of advance preparation as part of crisis recovery measures. Chien and Law [10] proposed the preparation of an emergency plan, wherein emergency teams should hold meetings regularly to evaluate the crisis and take immediate action if any crisis occurs. Mao et al. [26] indicated that the speed of disaster response is key in facilitating the recovery process. Rapid response is also tightly connected with the support of the government and industry funding [35]. Ghaderi et al. [36] introduced another rationale for successful crisis management, outlining the importance of knowledge and organizational learning and suggesting that tourism organizations should constantly survey their environment to acquire as much crisis knowledge as possible.

Response speed was also an important factor to be considered. Response speed was essentially dependent on the readiness of a destination; however, various destinations were not well prepared for crises/disasters [37,38]. Ritchie [39] mentioned that developing crisis/disaster management plans and implementing them quickly could help mitigate the effect brought about by a recent crisis/disaster. AlBattat et al. [40] found that media publicity, proactive contingency planning among industry stakeholders, government agencies, and local communities, and the urgent need for cooperation, coordination, and awareness-raising efforts could reduce the negative effects of dangerous occurrences on the hotel industry. Armstrong and Ritchie [35] argued that although advance preparation could be considered an effective measure for the recovery of the tourism industry from the crisis, the successful implementation of advance preparation was still largely dependent on the funding support of governmental and non-governmental organizations.

#### 2.2.2. Integrated Stakeholder Approach

Integrated stakeholder approach [41] was also considered a comprehensive and cost-effective method for the recovery of tourism industry after a crisis [25]. Because of the detailed aspects of this approach, in-time communication and effective marketing among relevant parties were considered to be effective measures in reducing the effects of infectious diseases on the industry. Thus, sharing the information gathered among relevant parties involved could be used to effectively handle the negative effects of crises/disasters on the industry. In terms of in-time communication, key elements of the approach advocated by Hayes and Patton [27] included the identification and assessment of risks, methods of taking appropriate action in disaster situations, and effective communication strategies. Ghaderi et al. [36] also mentioned the importance of an information communication management system. Ladkin et al. [42] considered the implementation and dissemination of marketing campaigns on recovery a crucial step. In particular, such campaigns should be performed at multiple levels, that is, from local to regional to national [42].

The post-crisis stage is considered more important than the pre-crisis and crisis stages because it plays a critical part in determining whether successful recovery is possible after crises/disasters. Previous studies have considered post-disaster marketing an effective measure for recovery. Different from traditional marketing methods, effective post-disaster recovery marketing should have an effective identification and communication tool with the target market using appropriate marketing plans. Thus, destination marketers should fully understand the changes in their target market after tourism crises/disasters, such as to redesign the methods of communicating information and marketing destinations and hotels. Walters and Mair [43] identified nine common types of post-disaster marketing, namely, usual marketing program, community readiness marketing, solidarity marketing, celebrity endorsement, confidence restoration, safety-focused marketing, curiosity enhancement, short-term discounts, and guest/visitor certificates. Moreover, specially designed packages may be prepared for the recovery of the industry. Huang and Min [44] suggested package marketing, which was a combination of flight tickets and accommodation offered after a crisis. Package marketing could also be extended to regional, cross-border, and multinational travel packages. Mckercher and Pine [45] claimed that promotional fares and short-term low prices were adopted to encourage tourist travel to southeast Asia after the outbreak of SARS in 2003, given that the region was the most preferred destination among Hong Kongers.

However, Chien and Law [10] suggested postponing promotional activities and avoiding unnecessary capital expenditures, such as temporarily closing hotel properties, to recover from a pandemic period. They also suggested lowering the hotel price to attract guests after such a period. Taylor and Enz [46] found that the recovery of the hotel industry after crises/disasters was reflected in two aspects, namely, increasing revenue and reducing costs. Increasing income mainly focuses on designing a marketing plan to raise hotel occupancy rates. By contrast, reducing costs mainly focuses on dealing with human resources and property management activities to reduce operating costs.

#### 2.2.3. Collaboration

Durocher [41] argued that public and private sector participation and cooperation among different parties were necessary to complete the crisis recovery process. Previous studies have indicated that the government had an important role in different stages of a crisis [29,47]. Before a crisis, the government needs to keenly identify and collect various information and data from previous tourism crises and design the measures to deal with different types of tourism crises. The government should also take the necessary measures for crisis prevention and conduct early warnings to avoid worsening the situation. The government should pay full attention to the crisis monitoring system, explore the root cause of a crisis, analyze and assess changes during a crisis, and establish a crisis management action system/team in coordination with all relevant parties in the society to implement timely and effective measures during a crisis [47]. Gu and Wall [47] suggested the formulation of preferential policies to reduce the economic burden on provinces suffering from a crisis by considering their local conditions. Lee and Warner [29] suggested that the Singapore government should take a series of measures to help laid-off workers. Such measures should include training and seminars for room attendants, tour guides, and event organizers to help in the recovery of the tourism industry after a crisis. Government measures for the post-crisis stage also include tax incentives or special import clauses to stimulate foreign investment, tax relief, and credit for hospitality organizations. The government should provide a summary of the crisis, take measures to restore the tourism market, such as restoring the tourist destination’s security image, and conduct targeted tourism promotions to restore consumer confidence in tourism by subsidizing damaged tourism companies and enterprises [10].

In summary, previous studies have identified three main approaches to the recovery of the hospitality and tourism industry after crises/disasters, namely, advance preparation, integrated stakeholder approach, and collaboration. The influence of the recent COVID-19 on the hospitality and tourism industry is large in scale (i.e., global). Although prior studies have suggested some recovery measures to address the effects of infectious diseases on the industry, whether the aforementioned measures were applicable to the recovery of the hospitality and tourism industry from COVID-19 remains unknown. Thus, this study adopted the three-stage crisis management framework from previous studies [34,48] and focused on crisis and post-crisis stages to investigate the influence of COVID-19 on the tourism industry.

## 3. Materials and Methods

### 3.1. Method

Previous studies adopted both the quantitative and qualitative research methods to investigate crises/disasters. For example, Wu et al. [30] proposed a factor modeling method to determine the influence of H1N1 on the hotel industry based on hotel occupancy rates in Hong Kong. They also provided useful suggestions for hotel practitioners about the dynamic change of hotel demand resulting from pandemics. Hung et al. [49] used a qualitative study method in their study to analyze data obtained from published official reports, statements, policy documents, on-site reports, and literature review search and discuss the effects of infectious diseases on the hotel industry. Lo et al. [50] conducted in-depth interviews with six hotel executives in Hong Kong to investigate the influence of SARS on hotels in the region and found that those hotels adopted various strategies to address the crisis at different stages.

**Figure 1:**
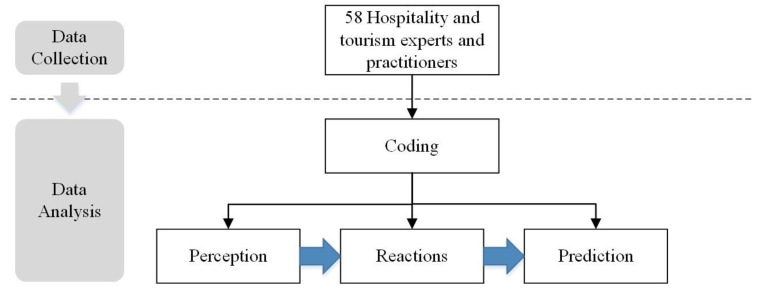
Research flow chart.

The present study adopted a qualitative approach, which is a method based on information exploration and analysis. Considering the novel nature of the COVID-19 pandemic, a qualitative research method was chosen to understand the effects of the COVID-19 pandemic on the hospitality and tourism industry and obtain comprehensive information from industry practitioners and experts [51]. The detailed flow chart is shown in Figure 1.

### 3.2. Data Collection

In-depth interviews were conducted with hotel and tourism managers and experts in China in order to formulate valuable suggestions for other countries and regions for reference, given that the first confirmed COVID-19 case was in Wuhan, China.

The present study adopted non-random purposive sampling, during which the sampling standard is determined according to the research question and research purpose, and whether the selected research samples can provide most information on a given topic [52]. In order to make the sample selected more referential, the present study selected influential hospitality and tourism experts from China’s tourism industry, such as tourism experts from major universities (e.g., Peking University) and the Tourism Research Institute. Hospitality and tourism experts were also recruited from National Tourism Administration, China Tourism Association, China Tourism Academy, and other national units. On the other hand, based on the division into the first-tier, second-tier, third-tier, fourth-tier, and fifth-tier cities, practitioners of tourist attractions in different types of cities were also selected. In addition, hotel managers were selected as interviewees according to different hotel types.

In total, 61 potential interviewees were approached, and 3 of them refused the interview request. As a result, 58 in-depth interviews, including 10 hotel managers and 10 hotel experts, 22 tourist attraction managers, and 16 tourism experts in China, were conducted via online communication channels (because of COVID-19) to understand the influence of COVID-19 on the hospitality and tourism industry. Data were collected from February to March 2020 through online personal interviews. Face-to-face interviews were limited because of the pandemic and the restrictions in different geographical locations. Specifically, telephone, WeChat voice call, video call, and other forms (e.g., email) were adopted. The average interview time for each interview was 20 min. Three main interview questions used in the interviews are listed as follows:

I. How do you evaluate the influence of COVID-19 on China’s hospitality/tourism industry?
II. From your perspective, what are the possible measures that can be used to recover China’s hospitality/tourism industry?
III. What are your predictions regarding the future development of China’s hospitality industry?

The researchers recorded the interview process with the consent of the interviewees and then converted the audio/video data into a transcript for further analysis.

### 3.3. Data Analysis

At the initial stage, word frequency analysis was conducted according to the in-depth interview transcripts. NVivo 11.0 (QSR International, Burlington, MA, USA) was then used to analyze the interview data of 10 hotel managers, 10 hotel experts, 22 tourist attraction managers, and 16 tourism experts. To ensure the rigor of the present study, researchers continued to analyze the remaining four interview transcripts after completing the coding process. The results indicated that no new information could be found from the aforementioned four interview transcripts, indicating that the content coded reached saturation.

In the present study, 58 well-organized original interview transcripts were imported to NVivo 11.0 software for coding purposes. Text data were coded from the bottom to the top. First, in the process of open coding, the researchers precoded the text data, clarified the meaning of words, sentences, and paragraphs, and carried out preliminary code names, marking them as initial nodes. Then, the researchers went through these nodes again to form a total of 25 nodes. After repeated comparison, analysis, and integration, the researchers summarized the 25 free nodes on 10 tree nodes. Finally, based on associative coding, the researchers further summed up “core genera” and finally formed three core codes.

## 4. Results

This section will be divided into subheadings. It should provide a concise and precise description of the experimental results, their interpretation, and experimental conclusions that can be drawn.

### 4.1. Demographic Profile of Interviewees

Table 1 shows the demographic profile of the interviewees. A total of 10 hotel managers, 10 hotel experts, 22 tourist attraction managers, and 16 tourism experts participated in the interviews. The gender ratio of hotel managers and experts is close to 1:1, while tourism managers and experts consist of a larger proportion of men, which is in line with the current gender share among China’s tourism practitioners. The 10 hotel managers were from different hotel groups, including a 4-star hotel, a 3-star hotel, economy hotels, and independent hotels in China. The 10 hotel experts were from various universities, a hotel association, and a hotel research association. A total of 22 tourist attraction managers were from scenic spots with different grades, which ranged from NA to 5A. Lastly, the 16 tourism experts were mainly from universities or tourism associations.

Table 2 further identifies the top 10 keywords obtained from the transcript, namely, “Epidemic situation”, “Hotel”, “Tourism”,” Scenic spot”, “Enterprise”, “Market”, “Loss”, “Affect”, “Service”, and “Recovery”. The findings show that “Hotel” and “Scenic spot” are the keywords that are mostly mentioned, reflecting the influence of the pandemic situation on hotels and scenic spots. The results also show that COVID-19 has affected the entire market of the industry and caused huge losses.

### 4.2. Perception of the Influence of COVID-19 on the Hospitality and Tourism Industry

**Figure 2:**
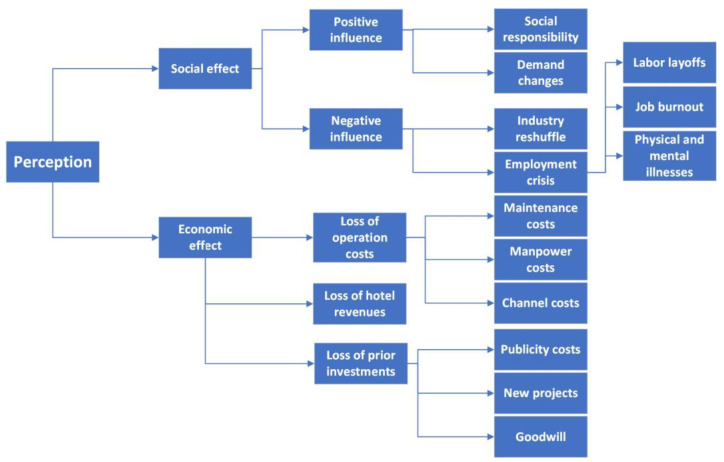
Perception of the influence of COVID-19 on the hospitality and tourism industry.

The results indicate that the influence of COVID-19 on hospitality and tourism is reflected in social and economic effects. Figure 2 shows the summary of the interviewees’ perception of the influence of COVID-19 on the hospitality and tourism industry.

#### 4.2.1. Social Effect of COVID-19 on the Hospitality and Tourism Industry

Regarding the social influence of COVID-19 on the hospitality and tourism industry, positive and negative effects were pointed out by hotel and tourism managers and experts. Positive impacts mainly include hotels’ social responsibility and changes in social needs. Specifically, hotels have been actively realizing social responsibilities, such as refunding reservations in full, providing accommodation for stranded and quarantined personnel, and conducting protective training for employees. For example, D13 proposed that the tourism industry should properly attend to tourists’ needs and give them humanitarian care. C8 said that hotels should provide catering and accommodation services for medical, quarantined, and stranded personnel. A3 proposed that, as a responsible enterprise, hotels should actively respond to relevant government policies. In terms of the changes in social needs, people have been paying more attention to health and sports due to the pandemic impact. For example, C7 pointed out that people are paying more attention to exercise, and it is expected that the national fitness products of tourism and sports would be popular.

Conversely, the main negative social effect that COVID-19 has brought on hospitality and tourism comprises the reshuffling of the hospitality or tourism industry and an employment crisis (e.g., labor layoffs, job burnout, physical and mental illnesses, and management issues). For example, B8 shared an observation that “as a public place, hotels have strong staff mobility and are prone to be affected by COVID-19. Hence, the hotel industry workers are facing a dilemma of fear of infection at work and losing money while not working.” B8 also expressed that “Hotel employees also showed symptoms, such as loss of enthusiasm for work, emotional irritability, and hopelessness for the future. Some employees even said that they would like to resign from the contract, and it is expected that post-traumatic stress syndrome will also occur even when COVID-19 pandemic period ends.” Moreover, B10 pointed out that “the number and proportion of employees who could get back to work after the crisis are still in doubt at present. In other words, whether hotel operation needs can still be met, whether there will be any personnel gaps, and whether the overall service skill proficiency of the staff could be maintained is not clear.” As tourism is an industry that is highly dependent on crowd gathering, tourist attractions were more affected than other industries by the pandemic. For example, C5 said that “There are human-resource-related problems in tourist attractions at present. Some small scenic spots have already dismissed cleaning and gardening workers with short-term employment due to operational pressure. Scenic spots are also faced with recruitment difficulties because of the pandemic. In addition, we found some employees had a serious slack mood due to unpaid leave and the training pressure after coming back to work.” Also, C9 pointed out that the cost of human resources in the scenic area is too high, and the reduction of staff is inappropriate; however, there were almost no tourists affected by the pandemic.

In summary, different groups have different perceptions of social influence, which is mainly due to the different positions of different groups. Hospitality and tourism managers think from the perspective of the enterprise itself. For example, the tourist attraction manager (C1) pointed out that the implementation of safety measures for pandemic prevention is conducive to shaping the image of the scenic spot and the hotel industry. Hospitality and tourism experts, on the other hand, tend to think from the perspective of the government. For example, tourism experts (D14) proposed that each scenic spot should increase its political compliance and resolutely control the spread of the pandemic.

#### 4.2.2. Economic Effect of COVID-19 on the Hospitality and Tourism Industry

In terms of the economic influence of COVID-19 on hospitality and tourism, hotel and tourism practitioners have expressed their ideas on the loss of operation costs, hotel revenues, and prior investments. Loss of operating costs is reflected in the losses of maintenance, manpower, and channel costs. Examples of the aforementioned necessary expenses include utilities, investment cost interest, loan interest, property costs, staff salaries, social insurance, venue rent, and compensation. A7 has expressed that “for the areas where Spring Festival is always the peak season, such as Sanya, Yunnan, and the ice and snow areas in the northern part of China, a lot of manpower and material resources have been invested before Spring Festival to prepare for the upcoming peak season, and the spread of COVID-19 resulted in heavy losses of the initial investment.” C5 said, “On the one hand, operational pressure has been increasing because before the epidemic is completely over, we have to bear huge labor costs and necessary facilities maintenance costs. On the other hand, operators in the scenic spots requested us to refund or reduce rental costs. Some small scenic spots are particularly difficult to operate, and some of them are facing bankruptcy pressure.”

In the aspect of revenue loss, the revenues of local and international hotels have been adversely affected through the entire pandemic period. For example, Ctrip’s “2020 Chinese New Year Tourism Forecast Report” previously predicted that 450 million people would have traveled during the Spring Festival 2020. However, during the COVID-19 pandemic outbreak, the customer service of Ctrip received 10 times more workload compared to normal over only a few days due to the millions of order changes during the Spring Festival 2020, including domestic and international travel and customized package tours. A1 also said that the economic losses brought about by COVID-19 to the hotel industry are extremely large. “In the statistics of our hotel (Sun Group Hotel) since COVID-19 pandemic started, the loss of hotel revenue from guest rooms and catering already reached 90%. The aforementioned situation is not only reflected in our hotel but also in the entire hotel industry, as the industry entered an unprecedented ‘cold winter’ caused by COVID-19.” Regarding the loss of prior investment, the initial investment loss includes the losses of publicity costs, new projects, and goodwill. A7 also mentioned that travel destinations preparing for Spring Festival 2020 lost their initial investment. C1 mentioned, “If you miss a golden season, the losses are certain. Compared with the same period last year, huge losses for this year can be estimated.”

Different groups have different perceptions of economic effects on the hospitality and tourism industry. Hospitality and tourism experts are more active in addressing economic impact. Most of them believe that the tourism industry will continue to generate revenue and that the pandemic is under control. For example, D4 pointed out that the recovery of the tourism industry after the pandemic, and concern over its recovery, is inevitable. D8 pointed out that the tourism market will rebound after the pandemic because of pent-up tourism consumption demand. Compared with hospitality and tourism experts, practitioners have a negative view of the economy. The reason may be because industry managers are at the forefront of the industry. Managers can experience the severe effects of the pandemic on the industry’s economy more directly. For example, C2 pointed out that tourist attractions are not simply experiencing losses; some small and medium-sized scenic spots may discontinue their operations. A5 also pointed out that the hotel has essentially no cash inflows and has to rely on previous capital to maintain operation.

### 4.3. Reactions (Possible Measures) to Reduce the Influence of COVID-19 on the Hospitality and Tourism Industry

**Figure 3:**
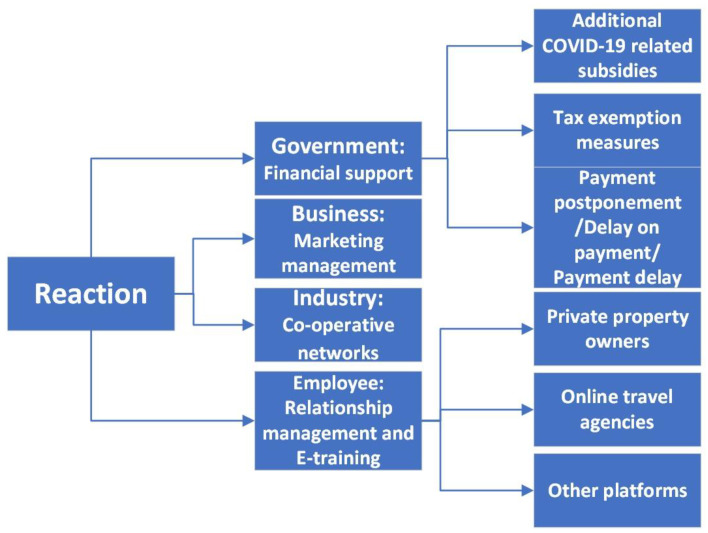
Reactions for reducing the influence of COVID-19 on the hospitality and tourism industry.

Hotel and tourism practitioners and experts have perceived government financial support, employee relationship management and e-training, business marketing management, and industry co-operation network as possible measures that can be used to reduce the influence of COVID-19 on the hospitality industry. In terms of the response, there was almost no difference observed between the answers from hospitality and tourism experts and managers. Hospitality and tourism experts have actively contributed their wisdom to the survival of the hospitality and tourism industry. Hotels and scenic spots have been actively seeking solutions to minimize losses. Figure 3 shows the possible measures suggested by the interviewees that can be used to reduce the influence of COVID-19 on hospitality and tourism.

#### 4.3.1. Government: Financial Support

Hotel and tourism practitioners expected that governments should, and probably must, provide additional subsidies and/or tax exemption measures. Hotel managers have also expected the government to postpone social security and provident fund payments for hotels. Specifically, with the COVID-19 pandemic under control, the government must gradually lift restrictions, implement drainage measures, and formulate preferential policies for the hotel industry after the pandemic period. Government policy support in the financial aspect must provide appropriate subsidies or appropriate tax exemptions, delays on social security and provident fund payments, coordination with banks to delay loan repayments, and reduction and exemption on interest rates, especially for hotels. In turn, hotels can alleviate their financial pressure through an established funding system from the central to the local level. For instance, A9 expressed that “the industry calls for preferential government policies, tax relief, and financial support. Governments at all levels have started to consider various measures to support small and micro enterprises.” B1 also mentioned that “re-examining the positioning and role of the hotel industry from the national strategic level and coordination with all resources from the perspective of integration of technologies can be considered to serve peace and emergency.” C3 believed that “The restoration of tourist attractions requires the relevant state authorities to assist and support tourism enterprises, including through financial support, tax reduction and exemption, delayed payment of personnel insurance, and so on, to ensure that tourism enterprises can survive this “tourism winter” smoothly.” C4 also thought that “The government should formulate policies to alleviate the difficulties that scenic spots face.”

#### 4.3.2. Employee: Relationship Management and E-training

Hotel and tourism practitioners and experts have also advocated for enterprise measures that can be strategically adjusted for post-COVID-19 pandemic plans. In other words, prevention, pandemic control, and a summary of the experience gained during this period are of great importance. Regarding the post-crisis recovery method, hotel and tourism practitioners and experts mentioned that hotels must communicate closely with their employees, support those who have difficulties, and provide timely assistance. Hotels must also create a safe working environment to make their employees feel safe at work after the COVID-19 pandemic period ends. Hotels must also enhance the quality and image of the entire enterprise, implement some activities showing corporate social responsibilities, conduct regular employee training, and improve the overall quality of employees for a fast and effective recovery after the COVID-19 pandemic period. B10 said that hotels must consider the following questions: “During the closure period, do hotel managers and employees contact each other and maintain a good relationship? Does the hotel staff group have cohesion and family warmth? Are WeChat, Microblog, and the official website still in operation, and are they constantly used to send greetings to their members? This special period is more powerful than any other period because if communications can be conducted with care between hotel employees and guests or members, more value can be gained.” Formulating an online course training plan must also be considered. By attending various courses from internal and external lecturers, employees can learn skills and improve their abilities even when they are isolated at home. In this manner, the overall quality of hotel employees can be improved. C2 also said that “Improving the infrastructure and safety and sanitation of the scenic spot with in-time communication online is considered a possible way of reducing the impact of the pandemic on tourist attractions.”

#### 4.3.3. Business: Marketing Management

Post-crisis marketing is of great importance. Hotels must consider marketing activities on community, online, or new media networks promptly in a timely manner, according to the changing market demand because of the COVID-19 pandemic. As hotel intelligence has become increasingly popular, hotel managers must also pay special attention to the digital marketing or transformation of hotels promptly, according to market changes. For instance, B2 expressed that “the increase in local bookings also means that there can be more opportunities for integrated consumption; for example, selling designed packages such as ‘hotels-catering’, ‘hotels-scenic spots’, ‘hotels-car rental’ and other product lines, formulating marketing strategies in advance, integrating hotel resources effectively, packaging attractive hotel products, and entering the market quickly.” C3 said, “If the scenic spot does not have enough money to upgrade its hardware, a small amount of money should be invested to upgrade and enrich tourism products, which could bring more fun for the tourists, to enhance the competitiveness of the scenic spot. It is also necessary to highlight the scenic spot for uninterrupted publicity and keep the scenic spot hot for customers.”

#### 4.3.4. Industry: Co-Operative Networks

Cooperation is considered a good way to reduce losses. Industry associations actively fulfill their responsibilities and help the tourism industry to survive by establishing a good communication network and practical measures. For example, industry associations issue notices to reduce or delay the rental fees for hotels. Industry associations also negotiate with online travel agencies and other platforms on the commission, credit card fees with UnionPay and/or other institutions, energy price adjustments (e.g., water and electricity), and health and pandemic prevention-related training. In addition, hotel associations in different regions also lead in organizing local small- and medium-sized hotels to form an alliance for hotel promotions, such as joint determination of hotel prices after the resumption of hotel operations, joint promotion activities, and mutual assistance in sharing human resources.

### 4.4. Prediction for Future Tourism Development after COVID-19

The results of the transcript analysis show that predictions for hospitality and tourism development after COVID-19 from the perspective of managers and industry experts are mainly reflected in two aspects, namely, hotel and tourism development forecasting and consumer behavior prediction and hotel and tourism product development. Hotel and tourism practitioners and experts have shared their recovery forecast in terms of the influence of COVID-19 on the hospitality and tourism industry. Specifically, they predicted that the industry will enter a new era. For instance, A7 expressed that “after the pandemic, the hotel industry will experience an explosive rebound in growth.” A9 also shared an observation that “as a strategic pillar industry of the national economy and a modern service industry required by consumers, we believe that the rainbow after the storm will make it possible for the hotel industry to develop by leaps and bounds after the pandemic period. It is predicted that the hotel industry will recover soon.” C15 also said, “In the face of the impact of the pandemic on scenic spots, operation capacity and management level of the scenic spot was tested. Perhaps some small scenic spots will be withdrawn after the pandemic. Furthermore, after the pandemic, people’s consciousness of health will be further enhanced. As a result, scenic spots should pay more attention to safety and sanitation and deliver the message to customers during promotion.”

Hotel and tourism practitioners and experts also predicted consumers’ behavioral and psychological changes. Specifically, they mentioned that because of the behavior developed during the COVID-19 pandemic, even after the pandemic is over, a remote office work system is still expected for some organizations considering the issue of travel cost reduction. Thus, business travel is expected to decrease. In addition, from the perspective of consumer psychology, consumers may develop higher requirements for the sanitation of hotel facilities after the pandemic period because of the continuous psychological fear influenced by pandemic prevention and control. Moreover, tourists may avoid unnecessary travel and develop suppressed expectations. For instance, A1 expressed that “after COVID-19 pandemic is fully controlled, it is expected that the hotel industry will enter a rare golden period; nonetheless, higher requirements of hardware facilities of hotels, services, and public safety should be considered.” B9 also said that “Since consumption needs, consumption patterns, and consumption characteristics of hotel guests are about to change, hotels need to take the aforementioned three aspects into consideration and provide more targeted and adaptive service products to hotel guests in the future.” D4 said, “After the pandemic, the recovery of tourism is inevitable, and the tourism industry will pay more attention to the quality of service. In addition, the tourism industry needs to constantly improve tourism products and business models in order to develop healthy and sustainable tourism.” C7 mentioned that “After the pandemic risk is lifted, people’s psychological expectations will be gradually restored, short-distance travel may increase, and national tourism consumption may increase as well. In addition, because of the pandemic, people have started to pay more attention to physical exercise and outdoor activities; hence fitness-related tourist products may become popular.”

**Figure 4:**
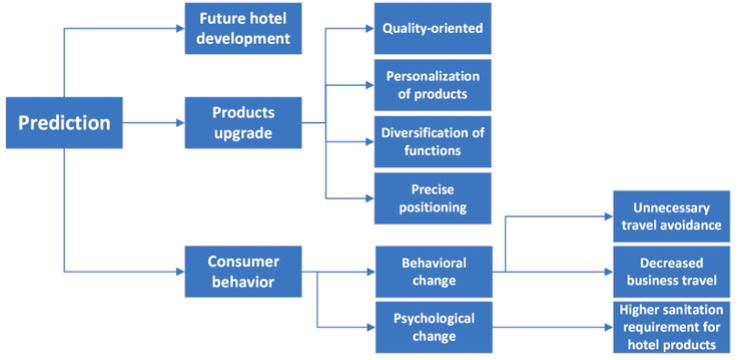
Prediction of hospitality and tourism development after COVID-19 pandemic.

Hotel and tourism practitioners and experts also identified a change in the upgrade and type of products. Specifically, they stated that consumers will pay increasing attention to the quality of products. Some enterprises may be closed, reorganized, transformed, and upgraded due to the COVID-19 pandemic. The upgrade of products is reflected in the personalization of products, diversification of functions, and precise positioning, which can be perceived as a development direction for hotels in the future. For instance, A5 expressed that “personalization of products, diversification of functions, and accurate positioning may be development directions for hotels in the future. Take e-sports/professional computer-gaming hotel for example. In the face of the depression caused by the outbreak of COVID-19, three newly opened hotels for e-sports/professional computer gaming not only have good sceneries, occupancy rate, and average room price, but revenue per available room sets a new record with annual revenue, although it may be related to the closure of internet cafes.” The upgrade of hotel products is also reflected in the quality and hygiene of hotel guest rooms. The hotel industry will face a prolonged downturn for approximately one year and then recover slowly. Then, new hotel investment behavior will decrease. According to B2, “with consumer demand showing a trend of rejuvenation, personalization, and diversification, the hotel industry faces unprecedented challenges in its single product line and traditional marketing mode.” Figure 4 illustrates the prediction of future hospitality development from the perspective of hotel managers and hospitality experts. C15 believed that “People’s pursuit of health will be further enhanced, hence outdoor mountain scenic spots may be attractive for tourists.”

In conclusion, after COVID-19, China’s tourism development is expected to focus more on product innovation and quality upgrades. The COVID-19 pandemic has sounded a wake-up call for the global tourism industry. Many ongoing crises are affecting the tourism industry, such as climate change [21]. The development of tourism after the pandemic will be different from that before the pandemic, and sustainable tourism will become the main theme of development. For example, Freya pointed out that under the threat of climate change, ecological protection must become a high priority for tourism after the pandemic [53]. The tourism industry also needs sufficient resilience to deal with crises [54].

## 5. Discussion

Theoretically, the present study contributes to the expansion of the crisis management model. The present study also contributes to the identification of the influence of COVID-19 on the industry and possible measures that can be used to reduce this influence based on the framework of crisis management. Specifically, this study focuses on the crisis and post-crisis stages by investigating the effects of COVID-19 on the hospitality and tourism industry. This research also builds on previous studies by providing possible measures that can be used to reduce such influence. The interviewed hotel and tourism practitioners and experts have predicted the future development of Chinese hotels affected by COVID-19. The findings also contribute to recent research on the influence of COVID-19 on the industry.

Practically, this study provides feasible suggestions that can be used by hotel and tourism practitioners in assisting the recovery of the hospitality industry during the COVID-19 pandemic period and the further development of Chinese hotels after the pandemic period. In addition, the pandemic situation is still not stable, and the assistance measures for the tourism industry need to be continued. Hotel and tourism practitioners in other countries and regions can also refer to the measures identified in the present study, such as increasing government support, industry alliances, strengthening marketing, and paying attention to employees.

Although the COVID-19 pandemic in China has been essentially brought under control, the pandemic has not yet ended and still poses a threat to many countries in the world. As mentioned by the hospitality and tourism practitioners and experts, although the COVID-19 pandemic brought many challenges for the hospitality and tourism industry, it promoted the digital transformation of the hospitality and tourism industry and accelerated the digitization of life, work, and learning in the whole society. Specifically, the digitalization of tourism has been accelerated by the increasing demand for contactless services. Virtual tourism has become a practical option for mass tourism during COVID-19, and it could possibly replace mass tourism after the pandemic [55]. Even after COVID-19, people may still continue to socially distance and reduce their mobility, which will increase the demand for Augmented Reality (AR) [56]. New business forms/models, such as cloud entertainment, cloud live broadcasting, and cloud exhibition have emerged. In addition, digital, cultural, and tourism consumption promoted by online tourism, virtual scenic spots, and online museum smart tourism have become new hot spots and trends for the tourism industry. Furthermore, forecasts from the hospitality and tourism professionals, including experts and practitioners, that hotel and tourism products will be upgraded and types changed, were gradually confirmed.

## 6. Conclusions

### 6.1. Concluding Remarks

**Figure 5:**
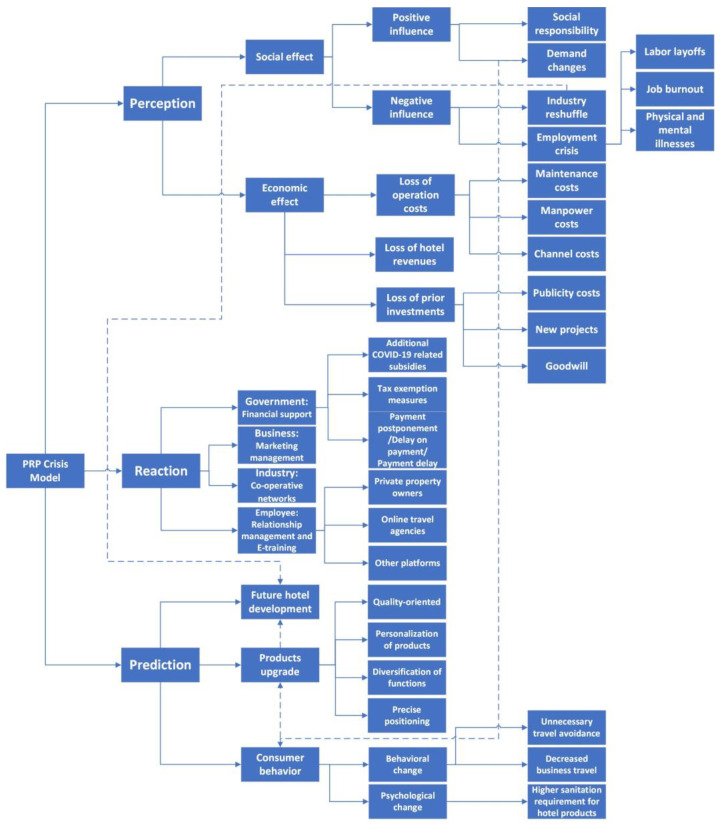
Proposed PRP crisis model for the hospitality and tourism industry.

Based on the three-stage crisis management framework, the present study identifies the influence of COVID-19 on the hospitality and tourism industry by conducting in-depth interviews with hotel and tourism practitioners and experts. The results show that the influence of COVID-19 on the industry is reflected mainly in social and economic effects. The social effect is mainly on corporate social responsibility, employee crises, and changes in the needs of tourists. The economic effect is mainly due to the huge losses caused by the pandemic, which has been confirmed by several academic researchers [20,23]. Cooperation between different stakeholders is key to reducing the effects of COVID-19 on the hospitality and tourism industry [40]. Possible measures that can be considered useful for the recovery of the industry in the future include government financial support, employee relationship management and e-training, business marketing management, and industry co-operation network. Hotel and tourism managers and experts have predicted the future of China’s tourism industry as affected by COVID-19, which is reflected in the behavioral changes of consumers and the upgrade of hotel and tourism products, particularly the quality and hygiene of guest rooms. The tourism industry in the future will be more resilient and sustainable [53,54]. Figure 5 shows the proposed Perception-Reaction-Prediction (PRP) crisis model for hospitality and tourism based on the findings of the present study.

Three differences are found between hotel managers and industry experts by comparing the ideas and thoughts on the early-stage of the COVID-19 pandemic of the hotel and tourism practitioners and experts. First, as hotel managers are the ones who are facing this crisis directly, they tend to be more nervous compared with tourism industry practitioners. Second, given that this pandemic is their first encounter with a crisis, hotel managers have no previous experience that they can use as a reference. Thus, compared with industry experts, hotel managers have expressed their anxieties when faced with such a crisis. Third, the focus of hotel managers is different from that of industry experts. The focus of hotel managers is more specific because of their strong practicality. For example, hotel managers are clear about the current situation of the market and understand the current weaknesses of hotels. Specifically, hotels urgently need government subsidies and tax reductions. By contrast, given that most of the hospitality and tourism industry experts have already encountered SARS during their academic career, they have been exposed to considerable literature related to different types of crises. Thus, when confronted with the recent COVID-19 pandemic, they tend to be more confident in dealing with such crisis from an academic perspective. Lastly, compared with the concerns of hotel managers, tourism industry practitioners should focus on crisis prevention measures and the situation as a whole and should be more concerned about the long-term development of the tourism industry, such as the time point where the industry could be considered fully recovered.

### 6.2. Limitations and Future Research

The present study has two limitations. The first limitation is that it only adopted a qualitative research method among hotel and tourism practitioners and experts. Future studies can adopt a quantitative research method or combine qualitative and quantitative research methods to obtain more comprehensive results, such as comparing the perception differences among hotel and tourism practitioners and experts statistically. Moreover, conducting interviews with public sectors can be considered. The second limitation is that the empirical findings of the present study are limited in scope because it used China as an example. Thus, empirical findings may not be fully applicable to other countries and regions. Future studies can examine the perception, response, and future development in other countries and regions affected by the crisis. A panel study could also be considered in the future because respondents may change their minds about the evolving situation.

## Figures and Tables

**Table 1:** Demographic profile of interviewees.

**Hotel Managers (A)**	**Gender**	**Organization**	**City**
A1.	F	Hotel Group	Beijing
A2.	M	Hotel Group	Beijing
A3.	F	Hotel Group	Beijing
A4.	F	3-Star Hotel	Beijing
A5.	M	Economy Hotel	Beijing
A6.	F	Economy Hotel	Beijing
A7.	F	Independent Hotel	Hunan
A8.	M	Independent Hotel	Suzhou
A9.	M	4-Star Hotel	Beijing
A10.	M	Hotel Group	Shanghai
**Hotel** **Experts (B)**	**Gender**	**Organization**	**City**
B1.	F	University	Beijing
B2.	M	Hotel Association	Shanghai
B3.	M	University	Beijing
B4.	M	University	Beijing
B5.	F	University	Beijing
B6.	F	University	Beijing
B7.	F	University	Beijing
B8.	F	University	Beijing
B9.	M	University	Beijing
B10.	M	Hotel Research Association	Sichuan
**Tourist Attraction Managers (C)**	**Gender**	**Scenic Spot Grade**	**City**
C1.	F	5A	Guiyang
C2.	M	4A	Wuhu
C3.	M	4A	Beijing
C4.	F	5A	Enshi
C5.	M	5A	Yongcheng
C6.	M	NA	Qiannan Buyi and Miao autonomous prefecture
C7.	M	NA	Baoding
C8.	F	3A	Bengbu
C9.	F	3A	Beijing
C10.	F	5A	Beijing
C11.	F	4A	Liangshan Yi autonomous prefecture
C12.	F	5A	Tianjin
C13.	M	4A	Jincheng
C14.	F	5A	Baoding
C15.	F	NA	Beijing
C16.	M	5A	Tangshan
C17.	M	4A	Beijing
C18.	M	5A	Zaozhuang
C19.	M	5A	Linyi
C20.	M	4A	Kaifeng
C21.	M	4A	Baishan
C22.	M	4A	Ningbo
**Tourism Experts (D)**	**Gender**	**Organization**	**City**
D1.	M	University	Beijing
D2.	M	Tourism Research Center	Beijing
D3.	M	Tourism Planning Institute	Beijing
D4.	M	Tourism Enterprises	Beijing
D5.	M	Special Experts in Cultural Industry of China Economic Net	Beijing
D6.	M	University	Beijing
D7.	M	Tourist Association	Beijing
D8.	F	University	Beijing
D9.	M	University	Guangzhou
D10.	M	University	Lanzhou
D11.	M	Chinese Academy of Social Sciences Tourism Research Institute	Beijing
D12.	F	University	Beijing
D13.	M	University	Beijing
D14.	M	Tourism Research Institute	Beijing
D15.	M	World Federation of Tourism Cities	Beijing
D16.	M	World Federation of Tourism Cities	Beijing

**Table 2:** Word frequency analysis.

Keywords	Frequency	Weighted Average Percentage (%)
1. Epidemic situation	245	4.56%
2. Hotel	225	4.18%
3. Tourism	168	3.12%
4. Scenic spot	157	2.92%
5. Enterprise	68	1.26%
6. Market	59	1.10%
7. Loss	59	1.10%
8. Affect	53	0.99%
9. Service	49	0.91%
10. Recovery	49	0.91%
